# Supplementary material for: Body Composition Evaluation and Clinical Markers of Cardiometabolic Risk in Patients with Phenylketonuria
Source: Nutrients. 2023 Dec 18;15(24):5133. doi: 10.3390/nu15245133 (PMC10745907; doi:10.3390/nu15245133)
Supplement: Supplementary file 1 [file nutrients-15-05133-s001.zip › nutrients-2702578-supplementary.pdf]

3.2.8. Comparison among techniques to evaluate quantity and quality of adipose and muscle tissues in patients with PKU.

Table S1. Advantages and disadvantages of measuring body composition using simple and predictive measurements, two component and electromagnetic techniques.

METHOD	ADVANTAGES	DISADVANTAGES
**DXA**<br>(Dual energy X ray absorptiometry)<br><br>Two component technique, special imaging modality<br>[149]<br><br>Assumption: model based on constant attenuations of fat free mass (FFM) and fat	Developed in the 1980s now widely available<br>Densiometric technique<br>Measures whole and regional body (arms, legs, trunk, head)<br>Quick<br>Low radiation<br>Accessible for children<br>Developed to measure bone mineral mass (BMM), and soft tissue composition. These are calculated from the differential absorption of X- rays of two different energies.<br>DXA measures depth and composition of soft tissue generating measurements of fat and lean body mass<br>Calculation makes assumptions for overlying soft tissue, values of fat and fat free mass (FFM), using instrument specific algorithms<br>Best at measuring regional (limb) lean mass which can be used to assess if weight loss is accompanied by changes in lean mass as well as fat mass<br>Body composition via DXA can be normalised for height using the fat mass index (FMI) defined as the total DXA fat mass normalised by height squared. FMI has the advantage over body mass index (BMI)for defining obesity status since it is independent of lean mass status [150,151]<br>DXA provides precise body composition analysis with a low radiation dose and, in children and adults, a good correlation with bioelectrical impedance analysis, skinfold thickness measurements and underwater weighing [152-155]	2-dimensional assessment<br>DXA results need to be interpreted with care in children<br>Normal reference data lacking<br>DXA predicts rather than measures soft tissue composition in pixels that contain bone, affecting the trunk data in particular<br>The accuracy of DXA for soft tissue composition is influenced by gender, body size and body composition. Although the data is robust to provide direction of trends<br>Fan beam machines are faster but have greater radiation which might be a disadvantage for healthy children<br>Prodigy machines uses a combination of fan and pencil beam measurements<br>Machines give different results GE Lunar has a bone mineral density (BMD) 16% higher than Hologic machines may be a problem if a subject has a scan on a different machine<br>Important when scanning children to know the source and number of children forming the reference database for the machine used in order to evaluate how appropriate this is for the child being scanned<br>Data must report the machine make and model and software version used if comparisons are being made<br>Limited use for measuring baseline fat mass or longitudinal changes in fat mass since measurements are known to be biased by body size (thickness).<br>Large/ obese individuals present two challenges length, width and rated load capacity may exceed the table dimensions- some may be too tall or wide to complete measurements. Although off set scanning can be used to obtain data in this set of patients
**Anthropometrics**<br><br>Measuring weight, height, BMI (body mass index), waist circumference, skinfold thickness (tricep, subscapular, suprailiac)<br>[156]	Easy to monitor weight, height, skin fold thickness, sub scapular thickness and waist<br>Cheap<br>Accessible<br>Non invasive<br>Quick, simple<br>Portable<br>Most age groups can be measured including young infants	BMI does not provide information on the proportions of fat and lean mass [167]<br>For children (most in obesity) there is a wide variability in the relative level of fat for a given BMI value<br>BMI is a proxy for nutritional status which is correlated with lean mass, skeletal muscle mass, fat mass and bone mass but is unable to differentiate between them<br>BMI is used as an index of relative weight often expressed as SDS to consider age and sex. In adults it can be used as a predictive outcome for type 2 diabetes

	**BMI** (Body mass index)<br>BMI calculated as weight / height 2 is used as an index of relative weight and expressed as standard deviation scores (SDS) accounting for age and sex. In adults BMI is predictive of clinical outcomes e.g., type 2 diabetes, but this is less clear in children and adolescents<br>BMI and its relationship with body composition is more controversial (see disadvantages)<br>**Waist Circumference**<br>Waist circumference provides a simple measure of central fatness and may be more predictive of adverse outcomes such as lipid profile or insulin resistance that total fat<br>Waist circumference supported as a robust index of abdominal fat and useful index of visceral fat [156]<br>Decreases in waist circumference in children have been linked to decrease in cardiovascular risk profile [157-159]<br>Waist circumference is increasingly advocated as an additional tool for identifying those at greatest risk of excess weight [160-162]<br>Waist : hip ratio in adults is associated with morbidity after adjustment for relative weight and similar findings in children [163]<br>Reference data available in the UK in children from 1988 [164] and international measurements published In 2020 [165]<br>**Skinfold thickenss measurements**<br>Skinfold thickness measures 'fatness', measuring subcutaneous fat depots<br>Intraobserver and interobsever error are low compared to between subject variability but in obese children accuracy and precision are poorer[166]<br>Skinfold and BMI are useful as a baseline and longitudinal measurement of adiposity<br>Best use of skinfold thickness data is as raw values where they act as reliable indices of regional fatness<br>Equations have been derived for the prediction of per cent fat or body density from skinfold thickness measurements	however, its predictive value for children and adolescence is less clear. Relation to body composition is less clear<br>BMI may be misleading in hospital patients where malnourished children have an increase in relative body fat and severe decrease in lean tissue<br><br>Weight relative to height and body mass index have poor sensitivity for monitoring response to treatment<br>Weight is a poor proxy for lean mass, and diseases can alter energy utilisation<br>In adults BMI has a U-shaped association with mortality whereas body fat has a linear association<br>Skin fold (raw data) assumes a constant protein content<br>Skin fold equations have no reference data, they are quick and simple but there are no population data and poor accuracy in individuals and groups<br>Skin fold equations may not be valid in some populations and this approach is unsuitable for longitudinal comparisons<br>Waist does not measure internal visceral fat<br>Skinfold thickness measurement rank individuals in terms of relative fatness or assess the size of specific subcutaneous fat depots<br>Predictive equations confound accurate raw values with predicted error
**BIA** (Bioelectrical impedance)<br><br>BIA is a predictive model, using the assumption that the conductor has a homogeneous composition, a fixed cross-	Predictive technique<br>Simple and quick<br>Safe<br>Non invasive<br>Portable<br>Measures impedance of the body to a small electric current. This	Predicts body composition, by regression equations<br>Uses various assumptions and provides a crude value for body composition<br>Analysis is unsuitable to predict body fatness as the technique only measures FFM [174]<br>Relation between bioelectrical data and TBW is influenced by the age range investigated

sectional area and uniform distribution of current density<br><br>Assumption is made on conductivity measuring body water. Based on the theoretical relation between the volume of a conductor and its electrical impedance [168-173]	model treats the body as a single cylinder, with measurements made between electrodes placed on the wrist and ankle<br>Adjustment of bioelectrical data for height allows estimation of total body water (TBW). Using predictive equations TBW is measured and converted to FFM<br>Body composition allows functional investigations, measures of cellular function via bioelectrical impedance<br>Bioelectrical impedance has the potential to measure regional body composition, but currently its value in routine clinical paediatrics is limited<br>If used to monitor individuals over time it can indicate the direction but not the magnitude of changes in lean mass<br>BIA shows to be very reliable over repeated trials and for repeated measurements<br>Segmental measurements of limbs or torso avoid the variability of body build and multifrequency measurements provide additional information about water distribution, but more data is needed on these types of measurements<br>Single frequency BIA has a high precision providing electrode placement is consistent and can measure short term changes in TBW in individuals	Published BIA equations are population specific with errors estimated to be ± 8% fat<br>Clinical difficulties such as oedema may complicate the results<br>The cross-sectional area of the body is not constant and the impedance varies with the amount and distributions of body tissues and fluids<br>Data is difficult to interpret in the absence of reference data<br>Factors affecting measurements include electrode placement, hydration status, food intake, exercise and temperature<br>Instruments must provide a constant current and be able to accurately detect resistance of the body to the current<br>Regular calibration is essential<br>Position of the arms can affect readings abducting the arm in a 2% increase in resistance , if the source and detector electrodes are placed closer than 4-5cm electrode polarization may occur and increase resistance<br>Limited information on Asian, black and native populations<br>Many paediatric equations have been published, choosing the right equation can be difficult
**QCT** (quantitative computed tomography)	QCT densiometric technique<br>Provides 3-dimensional assessment of structural and geometric properties of the skeleton<br>Not affected by body size	Higher radiation- unsuitable for healthy children and pregnancy
**pQCT** (peripheral quantitative computed tomography) [175-177]	Low radiation<br>Fast scanning time<br>Provides a 3-dimensional assessment of structural and geometric properties of the skeleton<br>Provides information on muscle and bone geometry	Not widely available, currently used as a research method
**MRI** (Magnetic resonance imaging) [178,179]<br><br>Uses strong magnetic fields and radio waves to produce detailed images of the body. The images are produced via hydrogen atoms in water or fat and builds up a picture allowing different tissue types	Imaging technique that estimates the volume rather than the mass of adipose tissue<br>Images based on spatial variations, uses hydrogen nuclei located in water or fat and uses these data to discern tissue type in imagining slices, which are put together to give tissue volume<br>MRI provides an estimation of regional body composition and is an accurate and viable approach for the estimation of intra-abdominal adipose tissue and a useful index of visceral fat	Difficult to compare results with other techniques<br>Patients with metallic devices excluded<br>Costly<br>Limited availability, operational complexity<br>In order to find the fat mass it is necessary to assume the fat content of adipose tissue and the density of fat. The latter is relatively constant, but the former is not<br>FM calculated by MRI is only present in adipose tissue, which other techniques quantify by a different entity from MRI, total FM versus adipose tissue mass

to be measured		

Abbreviations: BIA bioelectrical impedance; BMD bone mineral density; BMI body mass index; BMM bone mineral mass; DXA dual energy X ray absorptiometry; FMI fat mass index; FFM fat free mass; FM fat mass; MRI magnetic resonance imaging; pQCT peripheral quantitative computed tomography; QCT quantitative computed tomography; TBW total body water

Table S2. Summary of advantages and disadvantages of methods to assess body composition. DXA: Dual-energy X-ray Absorptiometry; BIA: Bioelectrical impedance analysis; CT: Computed tomography; MRI: Magnetic resonance imaging; ADP: Air-displacement plethysmography; US: Ultrasound imaging. 0: very low; +: some; +++: high; I: investigative/opportunistic.

**METHOD**	**Availability**	**Effort**	**Risk**	**Cost**
**Anthropometrics**	+++	+	0	0
**DXA**	I	+++	+	+++
**BIA**	+	+	0	+
**CT**	I	+++	+++	+++
**MRI**	I	+++	0	+++
**ADP**	I	+++	0	+++
**US**	+	+	0	+
